# Purinergic signalling links mechanical breath profile and alveolar mechanics with the pro-inflammatory innate immune response causing ventilation-induced lung injury

**DOI:** 10.1007/s11302-017-9564-5

**Published:** 2017-05-26

**Authors:** Djo Hasan, Paul Blankman, Gary F. Nieman

**Affiliations:** 1 Department of Adult ICU, University Hospital Erasmus MC Rotterdam, ’s-Gravendijkwal 230 3015 CE, Rotterdam, the Netherlands; 2 Department of Surgery, Upstate Medical University, 750 E Adams St, Syracuse, NY 13210 USA

**Keywords:** Extracellular ATP, Ventilation-induced lung injury, Diffuse alveolar damage, Purinergic signalling, CD39, CD73

## Abstract

Severe pulmonary infection or vigorous cyclic deformation of the alveolar epithelial type I (AT I) cells by mechanical ventilation leads to massive extracellular ATP release. High levels of extracellular ATP saturate the ATP hydrolysis enzymes CD39 and CD73 resulting in persistent high ATP levels despite the conversion to adenosine. Above a certain level, extracellular ATP molecules act as danger-associated molecular patterns (DAMPs) and activate the pro-inflammatory response of the innate immunity through purinergic receptors on the surface of the immune cells. This results in lung tissue inflammation, capillary leakage, interstitial and alveolar oedema and lung injury reducing the production of surfactant by the damaged AT II cells and deactivating the surfactant function by the concomitant extravasated serum proteins through capillary leakage followed by a substantial increase in alveolar surface tension and alveolar collapse. The resulting inhomogeneous ventilation of the lungs is an important mechanism in the development of ventilation-induced lung injury. The high levels of extracellular ATP and the upregulation of ecto-enzymes and soluble enzymes that hydrolyse ATP to adenosine (CD39 and CD73) increase the extracellular adenosine levels that inhibit the innate and adaptive immune responses rendering the host susceptible to infection by invading microorganisms. Moreover, high levels of extracellular adenosine increase the expression, the production and the activation of pro-fibrotic proteins (such as TGF-β, α-SMA, etc.) followed by the establishment of lung fibrosis.

**Electronic supplementary material:**

The online version of this article (doi:10.1007/s11302-017-9564-5) contains supplementary material, which is available to authorized users.

## Introduction

Diffuse lung injury can be triggered by several experimental conditions, amongst others: Severe pulmonary infection or extrapulmonary infection leads to acute pulmonary distress syndrome (ARDS) [1–5]; Mechanical ventilation with high inspiratory pressures and high tidal volumes of >30 ml/kg ideal body weight in animals with healthy lungs causes interstitial and alveolar oedema and diffuse lung injury (ventilation-induced lung injury (VILI)) [6]; And mechanical ventilation in surfactant-deactivated animals causes VILI even with low tidal volume (*LV*_T_) of 6 ml/kg [7]. On the other hand, experimental mechanical ventilation with airway pressure release ventilation (APRV) with relatively long inspiration time and very short pressure release period may protect the lungs [7–14].

It is clear that lung infection results in pro-inflammatory immune response. In addition, there is a general agreement that ventilation of inhomogeneous lung tissue and/or with a high tidal volume triggers a pro-inflammatory response [15–19]. The corollary to this is that reduction of tidal volume and return to homogeneous ventilation will reduce (but not prevent) this pro-inflammatory response [19]. However, the exact mechanism of ventilation-induced pro-inflammatory response that leads to VILI is still not fully understood [15–18]. Moreover, the combination of all mechanical breath parameters (known as the mechanical breath profile (MBP) airway pressures, volumes, flow rates and the duration at both inspiration and expiration) applied with each breath, which injure or protect the lung, is still not known.

In this review, we have searched the literature thoroughly to identify the (missing) link between the MBP, alveolar mechanics (i.e. the dynamic change in alveolar size and shape during tidal ventilation) and the inflammatory response that causes VILI.

## Alveolar mechanics, alveolar surface tension and VILI

Conventional mechanical ventilator settings consist of a static and a dynamic component. The static component is a continuous pressure or stress applied during both the inspiration and expiration and results in a static volume change or static strain. Strain is defined as a change in volume normalised by the original volume. The static stress and strain are referred to as positive end-expiratory pressure (PEEP) and the change in end-expiratory lung volume (EELV) normalised by the functional residual capacity (FRC) of the lungs, respectively. If the applied PEEP is zero, the EELV equals the FRC. Thus, EELV equals FRC plus PEEP volume. The dynamic component is the strain (i.e. tidal volume (*V*_T_) normalised by FRC or EELV) inflicted by the cyclic stress, i.e. the inspiratory pressure [20].

### Cyclic lung volume change with high energy load causes VILI in healthy lungs

Protti et al. determined the mean value of the global energy load calculated from the mean inspiratory capacity by means of whole-lung CT scan in 76 pigs with healthy lungs [6]. Mean inspiratory capacity was defined as the mean value of total lung capacity (TLC) measured at 45 cm H_2_O airway pressure minus the FRC measured at 0 cm H_2_O airway pressure. The global energy load during conventional mechanical ventilation comprised a static component due to lung volume caused by the applied PEEP and a dynamic component due to the applied inspiratory pressure. The lower and upper limit of the applied global energy load was set at the value of mean global energy load ±2SD (equivalent to an inspiratory capacity range of 30.9–59.7 ml/kg ideal bodyweight). Then, the animals were subjected to different levels of PEEP and inspiratory pressures. When the mean global energy load did not exceed its lower limit, lung damage was not observed and only 1 of the 29 experimental animals did not survive the experiment. In contrast, when the mean global energy load exceeded the lower limit of the global energy load, lung injury, pro-inflammatory cytokine production and death were observed in 26 of the 47 experimental animals. In these cases, lung damage was associated with high dynamic energy load rather than high static energy load [6]. In addition, the rate of the applied strain was associated with ventilation-induced lung oedema and lung injury [21].

### In surfactant-deactivated lungs cyclic lung volume change causes VILI even with low energy load

Remarkably, the threshold of the energy load above which lung damage occurred in healthy pig lungs was quite high (equivalent to a mean *V*_T_ of >30 ml/kg ideal body weight) [6], whereas a *V*_T_ of 6 ml/kg is already injurious in the surfactant-deactivated lungs [11]. This difference is clearly illustrated in the following experiment. In anaesthetised mechanically ventilated pigs, normal subpleural alveoli were filmed in real time using in vivo video microscopy. This study discovered that there was very little change in alveolar size in healthy lungs when ventilated with *LV*_T_ (6 ml/kg ideal body weight) or with *HV*_T_ (12 or 15 ml/kg ideal body weight). In contrast, after surfactant deactivation with a detergent as a model of ARDS, the alveoli became unstable: Many alveoli collapsed at the end of the expiration, and some of these collapsed alveoli were recruited during inspiration irrespective of the magnitude of the tidal volume. Alveoli that remained open at the end of the expiration became over-distended during the inspiration resulting in an inhomogeneous ventilation pattern in the lungs [22].

### Stabilization of the alveoli prevents VILI

**Fig. 1:**
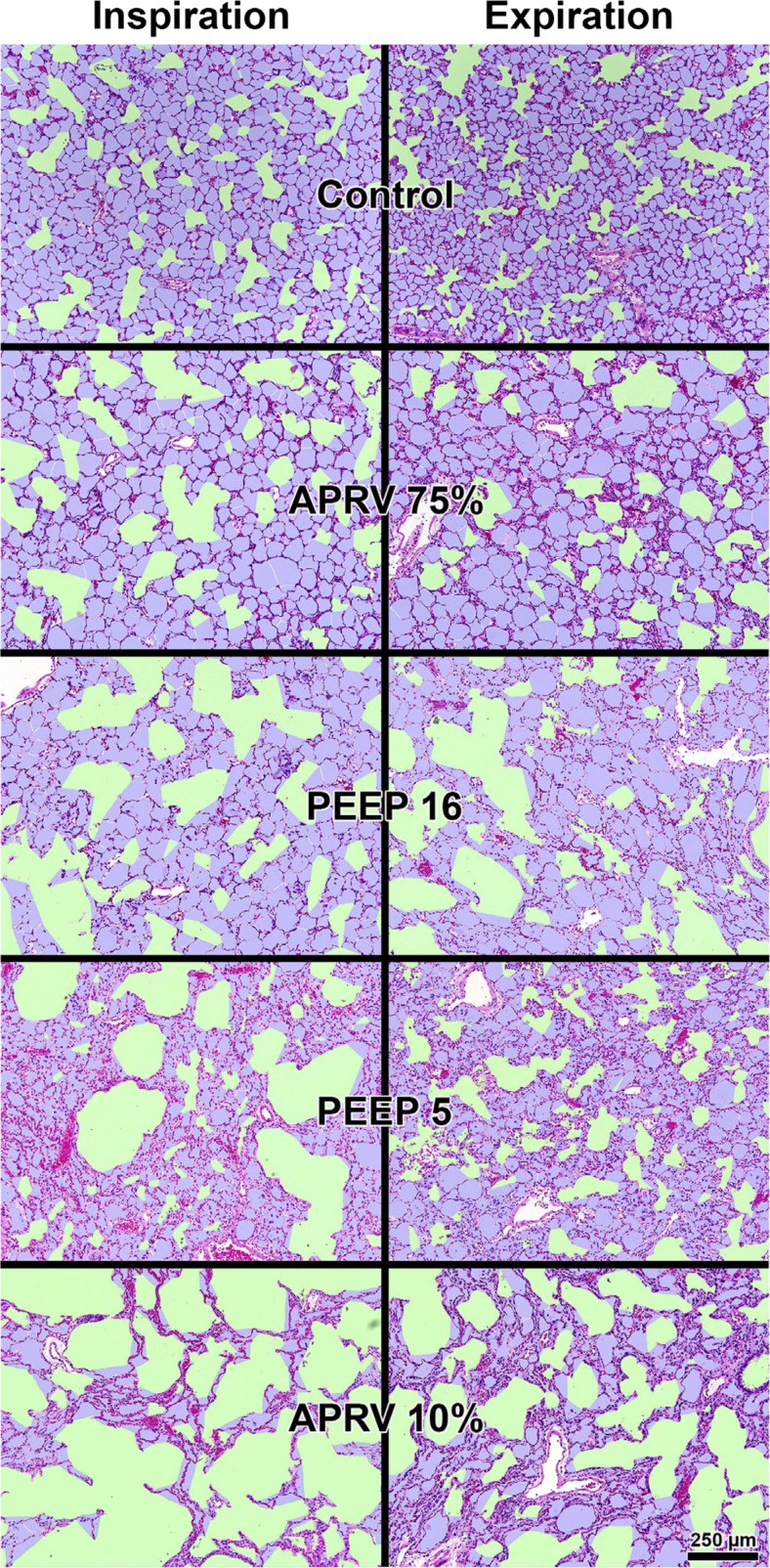
Photomicrographs of rat lungs. After *en bloc* excision of the lungs, one of the lungs was clamped and fixed in formalin at peak inspiration pressure and the other at end expiration pressure for histologic analysis. The respiratory bronchiole, alveolar ducts and alveolar sacs are *green*; the alveoli are *lilac*; and the alveolar walls are *magenta*. Healthy lungs ventilated with controlled continuous mandatory ventilation (CMV) with low tidal volume (≤6 ml/kg ideal body weight) and 5 cm H_2_O PEEP (control). APRV: surfactant-deactivated lungs with intratracheal instillation of a detergent ventilated with *T*_low_ being interrupted when the end-expiratory flow (EEF) reached 75% (APRV 75%) or 10% (APRV 10%) of the peak expiratory flow (PEF). (PEEP 5) Surfactant-deactivated lungs ventilated with the same settings as ‘control’ or with PEEP 16 cm H_2_O (PEEP 16) (see Table 1 for the mechanical breath profile data). Note that the highest alveolar duct surface area is reached during inspiration in APRV 10% followed by PEEP 5; the lowest alveolar duct surface area is observed in the control group. The highest alveolar stability (the smallest difference in alveolar duct surface area between inspiration and expiration) with the lowest microstrain is reached in healthy lungs (control) followed by APRV 75%. The lowest alveolar stability and the highest microstrain are seen in APRV 10% and PEEP 5. Microstrain is calculated as the change in length of the alveolar ducts between inspiration and expiration normalised by their original length. The difference between the control group and PEEP 5 is exclusively attributed to the surfactant function. APRV can either significantly increase (APRV 10%) or decrease (APRV 75%) the microstrain and the redistribution of air towards the alveolar ducts. Figure from Kollisch-Singule et al. [8] with permission

Bachofen and Schürch demonstrated with scanning electron micrographs that surfactant deactivation and consequently increased surface tension in the alveoli in detergent rinsed lungs increase the alveolar duct surface area at the expense of alveolar surface area as compared to normal lungs. The increased alveolar duct size is accompanied by a decrease in the alveolar diameter perpendicular to the axis of the alveolar ducts (alveolar wall compression) and by an increase in the alveolar diameter parallel to the axis of the alveolar ducts. Therefore, surfactant deactivation is accompanied by alveolar wall stretching [23]. This finding was confirmed in an experiment in anaesthetised rats by the group of Nieman. After *en bloc* excision of the lungs, one of the lungs was clamped and fixed in formalin at peak inspiration pressure and the other at end expiration pressure for histologic analysis. In this way, they were able to visualise the effect of surfactant deactivation (Tween-induced ARDS model) and mechanical ventilator settings on alveolar duct microstrain and the distribution of inspired air between the alveolar ducts and the alveoli (Fig. 1) [8]. Microstrain was calculated as the change in length of the alveolar ducts between inspiration and expiration normalised by their original length. As the increase in the alveolar duct size after surfactant deactivation is accompanied by alveolar wall stretching parallel to the axis of the alveolar ducts [23], alveolar duct microstrain is likely to be proportional to the alveolar wall stretching and alveolar wall strain.

Four ventilation strategies were tested by this group [8]: *LV*_T_ plus two levels of PEEP (5 and 16 cm H_2_O) and APRV with the expiratory termination set at either an end-expiratory flow (EEF) of 10% (APRV 10%) of the peak-expiratory flow (PEF) or an EEF of 75% of the PEF (APRV 75%) [24, 25]; the higher the EEF/PEF ratio (i.e. 75% as compared with 10%), the shorter the expiratory duration (see Table 1 for the MBP of the set ventilator modes). Previous studies have shown that APRV 75% stabilises alveoli preventing alveolar collapse during expiration, whereas an APRV 10% extends the duration of expiration resulting in alveolar collapse [12, 24, 25]. This study demonstrated that APRV 10% caused high microstrain and redistribution of air towards the alveolar ducts as compared with APRV 75%, which resulted in almost normal distribution of air (Fig. 1). The order of ventilation strategies that caused the most to the least redistribution of inspired air into alveolar ducts (inspiratory ratio *C*_a_/*A*_a_ in Table 2) and microstrain was as follows: APRV 10%, PEEP 5, PEEP 16, APRV 75% and control (Table 2). Obviously, the least injurious ventilator settings are those that mimic the microstrain and air redistribution levels in ventilated healthy lungs with intact surfactant function (control in Fig. 1). APRV 75% resulted in near normal microstrain and alveolar air distribution, which was very similar to that measured in normal lungs, even though APRV 75% was in a Tween-induced ARDS model (Fig. 1, APRV 75% and Table 2). In this high surface tension ARDS model, the lower the PEEP (PEEP 5 vs PEEP 16) and the longer the expiratory duration (10 vs 75%), the worse the microstrain and alveolar air distribution (Fig. 1, Table 2). This research group hypothesised that the mechanism by which APRV 75% was so effective at normalizing microstrain and alveolar air distribution was as follows: The extended time at inspiration (achieved by applying continuous positive airway pressure) recruited almost all alveoli, and the very short time at expiration prevented these newly recruited alveoli from collapsing. In contrast, APRV 10% resulted in the worst microstrain and air distribution since the high pressure and extended inspiratory duration opened alveoli but the excessively long expiratory duration allowed time for all of the newly opened alveoli to collapse (Fig. 1) [8]. One mechanism of this lung protection was that APRV 75% preserved surfactant function, measured by increased concentrations of surfactant protein-A (SP-A) and SP-B in the bronchoalveolar lavage fluid, as compared to controlled CMV with a *V*_T_ of 10 ml/kg ideal body weight [10]. Apparently, redistribution of air towards the alveolar ducts and the increase in microstrain in controlled CMV deplete pulmonary surfactant. This supports earlier works showing that mechanical ventilation can impair pulmonary surfactant function [26, 27].

**Table 1:** Mechanical breath profile (MBP) of the set ventilator modes

MBP	Ventilator mode				
	Control^a^	APRV 75%	PEEP 16	PEEP 5	APRV 10%
Plateau pressure (cm H_2_O)	N/A	36.7	31	22.8	36.7
Tidal volume (ml/kg)	6	10.9	5.68	5.34	17
PEEP (cm H_2_O)	5	N/A	16	5	N/A
Respiratory frequency (min^−1^)	55	29.2	55	55	26.9
Inspiratory time or *T*_high_ (s)	0.73	1.9–2.0	0.73	0.73	1.9–2.0
Expiratory time or *T*_low_ (s)	0.36	0.04–0.08	0.36	0.36	0.22–0.26

The columns are sorted according to increasing microstrain values as shown in Table 2. APRV: *T*_low_ being interrupted when the end-expiration flow (EEF) reached 75% (APRV 75%) or 10% (APRV 10%) of the peak expiratory flow (PEF)

^a^The values in the control column are set values of the mechanical ventilator. The values in columns of the remaining ventilator modes are measured MBP values [12]

**Table 2:** Microstrain and redistribution of the inspired air from the alveoli towards the alveolar ducts (ratio *C*_a_/*A*_a_)

Effects of MBP on the lung tissue	Ventilatory mode				
	Control	APRV 75%	PEEP 16	PEEP 5	APRV 10%
Microstrain	0.137	0.202	0.231	0.336	0.394
Ratio *C*_a_/*A*_a_ (inspiration)	0.33	0.65	0.81	0.9*	2.07*
Ratio *C*_a_/*A*_a_ (expiration)	0.35	0.51	0.79*	0.6	1.32*

The columns are sorted according to increasing microstrain values. Microstrain is calculated as the change in length of the alveolar ducts between inspiration and expiration normalised by their original length. The lowest microstrain is observed in the control group and increasing in APRV 75%, PEEP 16, PEEP 5 and APRV 10%, respectively. Table based on Table 2 in the article by Kollisch-Singule, et al. 2014 [8] with permission

*C*_a_ alveolar duct surface area, *A*_a_ alveolar surface area, *APRV 10%* the expiratory termination set at an end-expiratory flow (EEF) of 10% of the of the peak expiratory flow (PEF), *APRV 75%* the expiratory termination set at an EEF of 75% of the PEF

*p < 0.05 vs control

The protective effect of APRV 75% (APRV settings: *P*_high_ 16–22 cm H_2_O, *T*_high_ 4.5 s, *P*_low_ 0 cm H_2_O) on lung tissue injury was also demonstrated in anaesthetised pigs with extrapulmonary ARDS. In contrast to *LV*_T_ controlled CMV (*V*_T_ 6 ml/kg ideal body weight, RR 55/min, I/E 1:2, FiO_2_ 21% PEEP 5 cm H_2_O and guided by the ARDSnet protocol [28]), APRV 75% was capable of preventing the development of DAD while maintaining lung elastance low, despite the fact that APRV 75% generated similar transpulmonary pressure compared to *LV*_T_ [11].

## The characteristics of lung tissue damage in ARDS and VILI are pointing at the activation of the innate immune system

The histopathology of VILI is referred to as diffuse alveolar damage (DAD) and is very similar to the histopathology associated with experimental ARDS. Reportedly, in contrast to the animal models of ARDS [29, 30], histopathology of DAD is found in only one third to half of patients with clinical diagnosis of ARDS [31–33]. ARDS is caused by multiple primary disease conditions such as sepsis, haemorrhagic shock and pneumonia, and these patients will require mechanical ventilation. If set improperly, mechanical ventilation can cause a secondary VILI, which will aggravate the DAD caused by the primary disease. There are three distinctive stages in the histopathology of DAD [34, 35]: The early exudative stage initiates after 12 to 24 h with declining frequency to almost zero after 4 weeks, the late proliferative stage with increasing frequency reaching almost 100% after 4 weeks and the fibrotic stage after 1 week increasing gradually reaching about 60% after 4 weeks. In the first 12 to 24 h: capillary congestion, interstitial and alveolar oedema [34]. The oedema results mainly from capillary leakage of fluid and proteins [36]. After 24 h, the characteristic histopathology is the presence of hyaline membranes. These membranes are found adjacent to the alveolar ducts and alveolar walls consisting of eosinophilic structures with cell debris, plasma proteins and surfactant degradation products. The alveolar walls are oedematous and thickened and filled with myxoid matrix comprising fibroblasts and myofibroblasts (fibroblasts phenotype containing alpha smooth muscle actins (α-SMA)), slight infiltration of the interstitial space with lymphocytes, plasma cells and macrophages. A significant proportion of the alveolar epithelial type I (AT I) cells and a small proportion of the AT II cells are damaged and lost leaving the alveolar membranes exposed. This means that the physical barrier against pathogens—a component of the innate immunity—is disrupted. Self-renewal of the epithelium (AT I and AT II cells) is performed by the AT II cells [37] and is accompanied by AT II hyperplasia which appears in 3 to 6 days. Mild endothelial injury is also reported [34]. In the proliferative phase of DAD, hyaline membranes are no longer prominent. The characteristic histopathology in the interstitium is the proliferation of fibroblasts, myofibroblasts and AT II cells mixed with occasional inflammatory cells. This granulation tissue breaks through the basement membrane into the alveolar spaces. The proliferating AT II cells show metaplasia resembling squamous cell carcinoma [34]. These AT II cells migrate over the surface of the alveolar granulation tissue and the intraalveolar exudate establishing the so-called fibrosis by accretion [38]. In addition, fibrosis is also formed by collagen deposits in the process of the organization of the interstitial oedema [34]. These processes and the increase in the surface tension by the deficient surfactant production and function in the inflammatory process lead to the collapse of the alveoli, size reduction of the remaining open alveoli and dilated alveolar ducts [23, 38]. After 3 to 4 weeks, granulation tissue of the fibrotic process dominates the lung histology. Enlarged airspaces—most likely in the alveolar ducts [8, 23, 38]—are outlined by collapsed alveoli with thickened alveolar walls [34]. The percentage of fibrosis and the amount of interstitial tissue increase over time [39]. In addition, the normally non-muscular small arteries are being muscularised and muscular arteries show intimal and subintimal fibrous thickening. This development may end in pulmonary hypertension [34].

## Surfactant metabolism and physiological purinergic signalling in the lungs are required for the release of surfactant by the AT II cells

Since surfactant dysfunction plays a crucial role in the development of DAD, the regulation of metabolism and secretion of surfactant may contribute to the pathogenesis of DAD.

Surfactant lipids and surfactant proteins are synthetised through different pathways by the AT II cells in the alveolar walls. Surfactant protein (SP-A, SP-B, SP-C and SP-D) synthesis starts in the endoplasmic reticulum (ER). The surfactant protein molecules are transported from the ER to the Golgi complex. SP-A and SP-D are excreted directly to the extracellular space [40]. SP-A and SP-D (both are hydrophilic protein molecules) belong to the collectins, a family of calcium-dependent sugar-binding proteins (lectins) containing collagen-like sequences and carbohydrate recognition domains (CRDs). Collectins are soluble pattern recognition receptors (PRRs, components of the innate immunity) that can identify pathogen-associated molecular patterns (PAMPs) and several danger-associated molecular patterns (DAMPs) [41, 42]. SP-B and SP-C plus ATP-binding cassette subfamily A member 3 (ABCA3, a surfactant lipid transporter) are transported from the Golgi complex to the multivesicular bodies (MVBs). Fusion of MVBs and the lamellar bodies (LBs) leads to the proteolytic process of SP-B and SP-C to the mature form. The surfactant lipids are produced in the ER as well but transported directly to the LBs by means of the lipid transporter ABCA3. The hydrophobic proteins SP-B and SP-C are essential in the packing process of surfactant lipid molecules in the LBs [40].

**Fig. 2:**
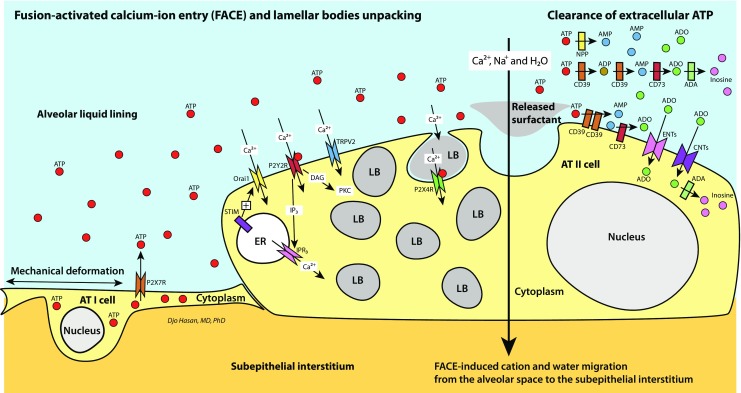
Schematic presentation of the fusion-activated Ca^2+^ entry (FACE). AT I cells release ATP to the extracellular space through the P2X7R (ATP receptor that can function as an ATP channel) provoked by mechanical deformation (compression or stretching) [43, 54–56]. The extracellular ATP activates the P2Y2Rs on the surface of the AT II cells in a paracrine manner. The G protein-coupled P2Y2 ATP receptor releases DAG and IP_3_ into the cytoplasm. DAG release leads to the activation of PKC-dependent pathway of fusion of LBs with the cell membrane. IP_3_ release results in the release of intracellular Ca^2+^ by stores that are sensitive to IP_3_ and Ca^2+^ entry from the extracellular space through several pathways (TRPV2 and STIM1/Orai1). Increased cytoplasmic Ca^2+^ level also promotes the fusion of the LBs with the cell membrane of the AT II cells. These two processes create a fusion pore causing the P2X4 ATP receptors in the membrane of the LBs to be exposed to extracellular ATP. Activation of these P2X4Rs by extracellular ATP strongly increases the local Ca^2+^ concentration to a much higher level around the membrane of the fused vesicles (FACE). FACE promotes a significant expansion of the fusion pore resulting in surfactant release by the AT II cells (LBs unpacking) and is accompanied by the FACE-induced cations and water migration from the alveolar space to the subepithelial interstitium. Clearance of the ATP molecules from the extracellular space occurs through the stepwise conversion by ecto-enzymes or by soluble extracellular enzymes (CD39 and CD73) to adenosine (ADO). ADO is returned to the cytoplasm through ENTs or CNTs and converted by ADA to inosine in the cytoplasm or converted by soluble ADA in the extracellular space. *AT I* alveolar epithelial type I cell, *AT II* alveolar epithelial type II cell, *ER* endoplasmic reticulum, *LB* lamellar body, *DAG* diacylglycerol, *PKC* protein kinase C, *IP*_*3*_ inositol triphosphate, *IP*_*3*_ *R* inositol triphosphate receptor, a membrane bound glycoprotein complex functioning as a Ca^2+^ channel sensitive to activation by inositol triphosphate, *TRPV2* transient receptor potential cation channel subfamily V member 2, a non-selective cation channel, *STIM1* stromal interaction molecule 1, a calcium sensor, *Orai1* calcium release-activated calcium channel protein 1, a calcium selective ion channel, *CD39* nucleoside triphosphate diphosphohydrolase 1 (NTPD1), *NPP* nucleotide pyrophosphatase/phosphodiesterase, *CD73* 5′-nucleotidase (5′-NT), *ADA* adenosine deaminase, *ENTs* equilibrative nucleoside transporters 1 and 2, *CNTs* concentrative nucleoside transporters 1 and 2

Under physiological conditions, mammalian cells contain high concentrations of ATP (5 to 8 mM) and ADP [43]. In the extracellular space, ATP can be detected in much lower (nanomolar) concentrations around resting cells [44]. Certain conditions may cause the release of ATP. In cell stress conditions such as an infection, ATP can be released in a controlled manner through pannexin hemichannels (mainly Panx1) [45, 46], connexin channels (mainly Cx43) [47, 48], certain chloride channels (maxi-anion channels, volume-regulated anion channels (VRAC), cystic fibrosis transmembrane conductance regulator (CFTR), chloride channel (ClC) family and calcium-activated chloride channel (CACC)) [49] and P2X7 ATP receptors (P2X7Rs) [50, 51] to >1000-fold of the resting state levels to micromolar concentrations. In cell ischemia and cell necrosis, ATP is released massively and uncontrolled [43, 52, 53]. Infection (such as in ARDS due to infectious agents) induces the release of extracellular ATP by many inflammatory cells and tissue cells (such as the AT I cells) [43, 52, 53]. In non-infectious circumstances such as physiological mechanical deformation (stretch or compression during breathing or mechanical ventilation) of the AT I cells stimulates the P2X7Rs followed by the controlled release of ATP molecules through the P2X7R channel into the extracellular space (Fig. 2) [54, 57].

Recently, using real-time luciferin-luciferase bioluminescence imaging coupled with simultaneous infrared differential interference contrast imaging, Furuya et al. were able to produce real-time imaging of inflation-induced ATP release in the ex vivo rat lung. They observed that lung inflation induces ATP release into alveolar spaces and into pulmonary blood capillaries [58]. In this way, extracellular purine nucleotides (such as ATP) play the key role in the extracellular signalling (purinergic signalling) of many biological processes and can activate purinergic receptors—for example, the P2Y2R—on the cell membranes [52]. The extracellular ATP originating from the AT I cells, released through the P2X7Rs by mechanical deformation, activates the P2Y2Rs on the surface of the AT II cells. The P2Y2R is a metabotropic G protein-coupled ATP receptor (GPCR). Activation of the P2Y2R by ATP results in the release of diacylglycerol (DAG) and inositol triphosphate (IP_3_) into the cytoplasm. DAG activates the enzyme protein kinase C (PKC) that promotes the fusion of LBs with the cell membrane (Fig. 2). Cytoplasmic IP_3_ results in the release of intracellular Ca^2+^ by stores that are sensitive to IP_3_ and Ca^2+^ entry from the extracellular space through several pathways (TRPV2 and STIM1/Orai1). The increased cytoplasmic Ca^2+^ level also promotes the fusion of the LBs with the cell membrane of the AT II cells and creates a fusion pore causing the P2X4Rs in the membrane of the LBs to be exposed to the extracellular ATP (Fig. 2). Activation of these P2X4Rs by extracellular ATP strongly increases the local Ca^2+^ concentration around the membrane of the fused vesicles to a much higher level (Fig. 2). This process—referred to as ‘fusion-activated’ Ca^2+^ entry (FACE)—promotes a significant expansion of the fusion pore resulting in surfactant release by the AT II cells (LBs unpacking). Thus, the AT I cells induce the unpacking of LBs by the AT II cells in a paracrine manner (Fig. 2) [54, 55, 59, 60]. In addition, at a higher degree of stretch of the AT II in vitro (probably comparable to a deep sigh), ATP molecules can be released to the extracellular space and induce LBs unpacking in an autocrine manner [57].

FACE drives a trans-epithelial current of Na^+^ and Ca^2+^ from the alveolar lumen through the P2X4R in the fused LB membranes and the cytoplasm to the subepithelial interstitium. This is followed by a passive water resorption from the alveolar space to the subepithelial interstitial space. In other words, extracellular ATP induces extracellular excretion of surfactant to the alveolar space and reduces the quantity of fluid in the alveolar liquid lining maximizing the reduction of the surface tension of the alveoli (Fig. 2) [56].

The P2X7Rs that release the extracellular ATP molecules are not subject to a refractory period during mechanical deformation [57]. In rat glomerular mesangial cell culture, 72% of the cells show an increase in Ca^2+^ levels during a 2-min stimulus with 100 μM ATP through the P2Y2Rs. In contrast to the p2X7Rs, the P2Y2Rs start to desensitise within 1 min, and the sensitivity to stimuli reaches its minimum within 2 to 4 min [61, 62]. After a recovery period of another 4 min, only 38% of the cells respond to a second stimulus. The response following the second stimulus is much weaker than the first stimulus [62]. The P2X4Rs become desensitised faster than the P2Y2Rs (within a few seconds after a stimulus with ATP), and the sensitivity to stimuli reaches its minimum after 30 to 60 s [63, 64]. Therefore, vigorous mechanical deformation of the AT I cells may cause excessive extracellular release of ATP but is unlikely to cause massive LBs unpacking.

Clearance of the ATP and ADP molecules from the extracellular space is performed through the stepwise conversion of extracellular ATP molecules by ecto-enzymes or by soluble extracellular enzymes to adenosine (Fig. 2). These enzymes comprise nucleoside triphosphate diphosphohydrolase 1 (NTPD1 or CD39, conversion of ATP to ADP and ADP to AMP), nucleotide pyrophosphatase/phosphodiesterase (NPP, conversion of ATP to AMP) and 5′-nucleotidase (5′-NT or CD73, conversion of AMP to adenosine). Then, adenosine is converted by soluble extracellular adenosine deaminase (ADA) to inosine or is returned to the cytoplasm of the cells through the equilibrative nucleoside transporters (ENT1 and ENT2) and concentrative nucleoside transporters (CNT1 and CNT2). Inside the cells, adenosine is being further processed by ADA and purine nucleoside phosphorylase (PNP) to inosine and hypoxanthine, respectively, and by adenosine kinase (ADK) to AMP [43, 52, 53, 65]. Under normal conditions, extracellular levels of ATP are kept low by the controlled release of these nucleotides and by the clearance of these nucleotides by the soluble or ecto-enzymes CD39, NPP and CD73 (Fig. 2) [43, 52, 53].

There are several mechanisms to clear surfactant from the alveolar space: (1) a small fraction of the surfactant lipids is squeezed out during the respiratory cycles to the bronchiole [66]; (2) degraded surfactant lipids are phagocytised by macrophages which is depending on the cytokine granulocyte macrophage colony-stimulating factor (GM-CSF) signalling through the macrophages GM-CSF receptor CSF2R [40]; (3) SP-D and the G protein-coupled receptor 116 (GPCR116) are involved in the reuptake of surfactant into the AT II cells and the MVBs for recycling purposes. Some of the lipid molecules are taken up by the lysosomes for further degradation [40]; (4) phosphates from degraded lipids are returned to the AT II cells by means of the sodium-dependent phosphate transport protein 2B (coded by the transcription factor SLC34a2) [40].

Obviously, the loss of the alveolar epithelial cells in DAD results in reduced LB surfactant stores and reduced surfactant production. Moreover, surfactant production can be decreased by pulmonary infection [67] and by hyperoxia (ventilation with air with 98% oxygen content) [68]. Surfactant deactivation by extravasated serum proteins [69] and the conversion of the active to the non-active surfactant subfraction contribute to further surfactant impairment [27]. Loss of surfactant production is accompanied by the deficiency of SP-A and SP-D molecules. As discussed above, SP-A and SP-D are soluble PRRs; they are important components of the innate immunity for the recognition of PAMPs and several DAMPs [41, 42]. Therefore, deficiency of SP-A and SP-D weakens the immune defence against invading pathogens.

## Prolonged induced excess of purinergic signalling leads to pro-inflammatory immune response, immunodepression and lung fibrosis

The effects of purinergic signalling through the binding of nucleotides (ATP, ADP, UTP and UDP) and nucleoside (adenosine) to different receptors on the innate and adaptive immune system are presented in Table 3. This table clarifies the importance of purinergic signalling in the regulation of both pro-inflammatory and anti-inflammatory responses of the immune system and the fibrotic process in the tissues.

**Table 3:** Summary of the effects of extracellular nucleotides and nucleoside on the innate and adaptive immune system through different purinergic receptors

Effects of extracellular nucleotides and nucleoside on the innate and adaptive immune system through different purinergic receptors					
Row number	Receptor	Ligand [72]	Immune cell expression	Results of receptor signalling	Reference number
1	AdoRA1	Adenosine	Neutrophils	Promotes chemotaxis	[73, 74]
2			Neutrophils	Increases adherence to endothelial cells	[75]
3			Neutrophils	Inhibits TNF-α release	[76]
4			Neutrophils	At low concentrations, adenosine enhances FcγR phagocytosis and actin dynamics.	[77–79]
5			Neutrophils	Restores LPS-inhibited chemotaxis	[80]
6			Resting DCs (rDCs)	Inhibits vesicular MHC class I cross-presentation	[81]
7			Plasmacytoid DCs (pDCs)	Potent chemoattractants, reduces IL-6, IL-12 and IFN-γ release	[82]
8	AdoRA1 and AdoRA2A		CD39^high^ B cells (Bregs)	Promotes expansion and function of CD39^high^ B cells	[83, 84]
9	AdorA2A	Adenosine	Monocytes	Inhibits IL-12 and TNF-α release	[85, 86]
10			Neutrophils	Promotes chemotaxis	[74]
11			Neutrophils	Inhibits oxygen radical generation	[73]
12			Neutrophils	Inhibits upregulation of beta2 integrins or MAC-1 (CD11/CD18) and shedding of L-selectin by FMLP	[87, 88]
13			Neutrophils	Promotes Cox-2 and PGE2 release	[89]
14			Neutrophils	Decreases adherence to endothelial cells	[75]
15			Neutrophils	Decreases adherence to fibrinogen coated surfaces	[90]
16			Neutrophils	Inhibits TNF-α release and expression of mRNA of TNF-α, chemokines MIP-1α (CCL3), MIP-1β (CCL4), MIP-2α (CXCL2) and MIP-3α (CCL20)	[76, 91]
17			Neutrophils	At high concentrations, adenosine inhibits FcγR functions and actin dynamics	[77–79]
18			Neutrophils	Inhibits leukotriene (LTB4, LTA4) synthesis	[92–96]
19			Neutrophils	Inhibits degranulation and superoxide release or oxidative burst	[90, 97–100]
20			Neutrophils	Delays neutrophil apoptosis	[101]
21			Neutrophils	Inhibits autophagy suppressed apoptosis of neutrophils by blocking caspase-8, caspase-3 and PARP signalling	[102]
22			Macrophages	Inhibits LPS-induced TNF-α release	[104]
23			Endothelial cells	Reduces thrombin-induced permeability. Inhibits thrombin-mediated expression of VCAM-1, ICAM-1 an E-selectin. Inhibits thrombin induced increase of IL-6, HMGB-1; chemokines, MCP-1 (CCL-2), CXCL-1 and CXCL-3	[105]
24			Naïve T cells	Promotes the differentiation towards CD4^+^FoxP3^+^Lag3^+^ Tregs, inhibits Th1 and Th17 differentiation, inhibits IL-6 secretion and increases TGF-β secretion	[106]
25			Th1, Th2 and Th17cells	Reduces release of IL-2, IL-4, TNF-α, and IFN-γ	[107–109]
26			CD8^+^CTLs, Th1, Th2	Reduces release of IL-2, TNF-α, IFN-γ. Inhibits CD8^+^CTL and Th1 expansion to alloantigens.	[110]
27			CD4^+^ T cells	Inhibits TCR-mediated IFN-γ release	[111]
28			CD4^+^CD25^+^FoxP3^+^ Tregs	Increases number of Tregs and increases the expression of CTLA-4 receptor	[112]
29			CD4^+^CD25^+^FoxP3^+^ Tregs	Upregulates ecto-enzymes CD39 and CD73 expression accelerating adenosine generation from extracellular ATP	[112]
30			AdoRA2A knockout mice	Bleomycin-induced fibrosis is more severe and elevated TGF-β1 is higher than in wild-type mice	[113]
31	AdoRA2A and AdorA2B	Adenosine	Macrophages	Differentiation of monocytes towards M2 macrophages with VEGF and IL-10 release	[114–118]
32			Macrophages	Inhibits LPS-induced IL-6, MIP-2 and TNF-α release	[119, 120]
33	AdoRA2B	Adenosine	Neutrophils	Inhibits neutrophil recruitment and transmigration, release of TNF-α, IL-6, MIF-1α and IL-8	[121, 122]
34			Neutrophils	Inhibits superoxide generation	[123]
35			Neutrophils	Inhibits TNF-α release	[76]
36			Macrophages	Stimulates IL-10 release	[124]
37			DCs	Differentiation and maturation towards regulatory DCs: High level expression of angiogenic (VEGF), wound healing (IL-6), chemokine (IL-8), immune suppressing (IL-10) and tolerogenic (IDO) factors	[125]
38			DCs	Promotes Th17 differentiation via stimulation of IL-6 release	[126]
39			Bone marrow cells	Promotes differentiation towards CD11c^+^Gr-1^+^ DCs that promotes Th17 response	[127]
40			Myeloid cells in systemic bleomycin-induced pulmonary fibrosis	Myeloid cells AdorA2B knockout mice show a reduction in CD206 and arginase-1 (markers for M2 macrophages). 10-fold reduction in IL-6 and 5-fold reduction in hyaluronan (both linked to pulmonary fibrosis)	[128]
41			Mast cells	Increases IL-1β, IL-3, IL-4, and IL-8 and IL-13 release	[103]
42			B cells	Induces Ig-E release through IL-4 and IL-13 release by the adenosine-activated mast cells	[103]
43			Endothelial cells	Reduces endothelial permeability, ICAM-1, P-selectin and E-selectin (adhesion molecules)	[129]
44			Endothelial cells	Stimulates basic fibroblast growth factor (bFGF) and insulin-like factor-1 release	[130]
45			Bronchial epithelial cells (HBEC)	Increases IL-19 release	[131]
46			Monocyte (THP-1)	Increases TNF-α release through HBEC-released IL-19	[131]
47			Renal fibroblasts	Increases the expression of α-SMA, IL-6, TGF-β, CTGF and fibronectin (pro-fibrotic mediators)	[132]
48			AdoR2B knockout mice	Negligible effect on bleomycin-induced acute lung injury. Enhanced loss of barrier function	[239]
49			AdorR2B knockout mice exposed to systemic bleomycin	Substantial reduction of fibrosis and IL-6 production.	[239]
50	AdoRA2B and AdoRA3	Adenosine	Mast cells	Stimulates IL-8 (chemokine) and VEGF (angiogenic) release.	[133]
51	AdoRA3	Adenosine	Neutrophils	Synergistic AdorA3 and P2Y2R neutrophil chemotaxis through autocrine ATP release by pannexin-1, extracellular conversion of ATP to adenosine by the ecto-enzymes (CD39 and CD73), strategic translocation of the FPR, AdorA3, P2Y2, pannexin-1 receptors and CD39, Cd73 to the leading edge of the neutrophils. This results the in amplification of the chemoattractant gradient sensing and the self-generated gradients.	[134–140]
52			Macrophages	Play an important role in the chemotactic navigation towards apoptotic cells	[141]
53			Microglial cells and colonic epithelial cells	Suppresses LPS-induced TNF-α production.	[142, 143]
54			Anti-CD3-activated CD8^+^ CTLs	Reduces the expression of mRNAs coding for granzyme B, perforin, Fas ligand and TNF-related apoptosis-inducing ligand (TRAIL). Diminishes Nalpha-CBZ-L-lysine thiobenzylester esterase activity (enzyme with cytotoxic activity). Reduces IL-2 sand IFN-γ release.	[144]
55			AdoRA3 knockout mice exposed to intratracheal bleomycin	Increases eosinophil numbers and selective upregulation of eosinophil-related chemokines and cytokines. But decreases eosinophil peroxidase activity in the BALF.	[145]
56	P2X1R	ATP	Neutrophils and platelets	Promotes thrombosis and fibrinogenesis: Keeps circulating neutrophils in quiescent state, recruit neutrophil to the injury site, activate adhered neutrophils and platelets	[146]
57	P2X1R, P2X4R and P2X7	ATP	Naïve T cells	TCR stimulation results in the translocation of pannexin-1 hemichannels, P2X1Rs and P2X4Rs to the immune synapse. While the P2X7Rs remain uniformly distributed, this process is required to induce calcium entry, NFAT and release of IL-2.	[147]
58	P2X3R	ATP	Mast cells	Increases the expression of IL-4, IL-6, IFN-γ, TNF-α, RANTES and MIP-2. Increases the release of IL-6 and IL-13	Article retracted due to figure irregularities [148]
59	P2X4R	ATP	γδ T cells	Activates and upregulates TNF-α and IFN-γ release	[149]
60			Microglial cells	Promotes survival after LPS activation	[150]
61	P2X4R and/or P2X7R	ATP	Neutrophils, monocytes, macrophages, DCs, CD4^+^ T cells, CD8^+^ T cells, iNKTs, adenoviral infected macrophages and alveolar epithelial cells	Mediates NLRP3 inflammasome-dependent IL-1β and IL-18 secretion (signal 2, non-classical pathway), increase IL-6 production	[70, 151–157]
62			Matured peripheral T cells	High-dose ATP promotes apoptosis, cell death CD62L shedding (homing receptor for central T-cells) independent from the NAD^+^-induced ART2-P2X7 pathway	[158–161]
63	P2X7R	ATP	Monocytes	Induces MMP-9 and TIMP-1 release, fibrosis markers	[162]
64			M1 macrophages	Induces the release of 74 pro-inflammatory proteins detected by antibody protein array and 33 inflammatory proteins detected by LC-MS/MS	[163]
65			M2 macrophages	Induces the release of 21 anti-inflammatory proteins detected by LC-MS/MS	[163]
66			Macrophages	Enhances intracellular bacterial killing	[164]
67			Mast cells	Induces degranulation	[165]
68			Naïve NKTs	Facilitates NAD^+^-induced inhibitory signal through the ART2-P2X7 pathway resulting in non-functional NKTs	[166]
69			Activated NKTs	Facilitates NAD^+^-induced stimulatory signal through the ART2-P2X7 pathway resulting in functional NKTs with increased IFN-γ and IL-4 release	[166]
70			B cells	Induces shedding of IgE receptor (CD23) and CXCL16. Soluble CD23 sustains growth of B cell precursors, promotes B and T cell differentiation and drives cytokine release from monocytes. CXCL16 is a chemoattractant for lymphocytes.	[167, 168]
71			CD11c^+^CD103^+^ DCs	Mediates infection-induced rapid recruitment of CD11c^+^CD103^+^ DC subsets into the epithelial layer of the gut.	[169]
72			Naïve T cells	TCR stimulation triggers rapid release of ATP and upregulates P2X7 gene expression. Autocrine ATP stimulation through the P2X7R is required to for the TCR-mediated calcium influx, NFAT activation and IL-2 production.	[170]
73			T follicular B helper cells (Tfh cells)	Controls the number of Tfh cells in Peyer’s patches in the gut with high-affinity IgA responses to promote host-microbiota mutualism	[171]
74			CD4^+^CD25^+^FoxP3^+^ regulatory T cells (Tregs)	Facilitates NAD^+^-induced Tregs depletion through the ART2-P2X7 pathway	[172]
75			DCs	Increases CD80, CD 86, STAT-1 and P2X7R expression, IFN-β release and T-cells expansion. Reduces Tregs numbers.	[173]
76			AT I cells	Induces VCAM-1 shedding and neutrophil transmigration in acute lung injury.	[174]
77			Brain-derived type-2 astrocyte cell, mesangial cells	Stimulates TGF-β mRNA expression.	[175, 176]
78	P2Y1R and P2Y12R	ADP > ATP	Platelets	P2Y1R and P2Y12R synergistic action in thrombin-induced platelet activation.	[177]
79	P2Y2R	UTP ≥ ATP	Neutrophils	Synergistic AdorA3 and P2Y2R neutrophil chemotaxis (see under AdoRA3 above)	[134, 135]
80			Neutrophils and fibroblasts	Mediates recruitment of neutrophils into the lungs, proliferation and migration of lung fibroblasts and IL-6 production	[178]
81			Monocyte-derived DCs (moDCs), eosinophils	Promotes chemotaxis	[179]
82			Eosinophils	Induces VCAM-1 expression	[180]
83	P2Y4R and P2Y12	UTP ≥ ATP, ADP > ATP, respectively	Microglial cells	P2Y4R and P2Y12R synergistic action increases microglial chemotaxis	[181, 182]
84	P2Y6R	UDP > UTP ≫ ATP	Neutrophils	Induces neutrophil activation and extracellular trap formation	[183]
85			Monocytes (THP-1 cells)	Induces IL-1β release	[184]
86			Macrophages	Induces MCP-3 (CCL7) expression in response to necrotic tissue cells	[185]
87			Microglial cells	Facilitates phagocytosis	[186]
88			Microglial cells	Induces the expression of MCP-1 (CCL-2)	[187]
89			Microglial cells	Promotes phagocytosis	[188, 189]
90			Basophils	UDP promotes IgE-dependent degranulation	[190]
91			Tissue cells	Induces IL-1α, IL-8/CXCL8 and IL-6 release	[184, 191, 192]
92			Tissue cells	Induce IFN-β release	[193]
93	P2Y11R	ATP	Neutrophils	Inhibits neutrophil apoptosis	[194]
94			Neutrophils	Enhances chemotactic response	[195]
95			Neutrophils and moDCs	Induces maturation of the granulocytic progenitors and monocyte differentiation	[196, 197]
96			moDCs	Inhibits migratory capacity	[198]
97			moDCs	Induces IL-8 release	[199]
98			Monocytes	Autocrine differentiation towards M1 macrophages, induces IL-1β, IL-6, IL-12 and TNF-α release	[200]
99	P2Y12R	ADP > ATP	Monocytes	Increases monocyte adhesion	[201]
100			Vascular smooth muscle cells	Upregulates MCP-1 (CCL-2)	[201]
101			DCs	Increases antigen endocytosis with subsequent enhancement of specific T cell activation	[202]
102			Microglial cells	Induces movement of juxta-vascular microglial processes to close the injured blood-brain barrier (BBB) and microglial activation	[203, 204]
103			Microglial cells	Promotes migratory, inflammatory (TNF-αand IL-6 release) responses	[205]
104			Microglial cells	ADP-treated microglial cells induce CCL3 expression in activated T cells	[206]
105	P2Y13R	ADP > ATP	Red blood cells	Inhibits ATP release	[207]
106	P2Y14R	UDP > UDP-glucose	Neutrophils	Enhances chemotactic response through IL-8 dependent manner	[208, 209]

In general, extracellular ATP activates the immune system, and extracellular adenosine exerts a depressive action on the immune system and possesses pro-fibrotic properties [70, 71]

*AdoR* adenosine receptor, *TNF-α* tumour necrosis factor alpha, *FcγR* receptors belonging to the immunoglobulin superfamily, *IFN-γ* interferon gamma, *IFN-β* interferon beta, *MAC-1* macrophage-1 antigen comprised CD11b (integrin αM) and CD18 (integrin β2), *PARP* poly ADP ribose polymerase, *FMLP N*-formylmethionyl-leucyl-phenylalanine, a chemotactic factor, *COX-2* cytochrome C oxydase polypeptide II, *PGE2* prostaglandin E2, *MIP-1α* macrophage inflammatory protein 1 alpha (MIP-1α = CCL3: chemokine ligand 3), MIP-1β (CCL4), MIP-2α (CXCL2 chemokine CXC motif ligand 2) and MIP-3α (CCL20), *RANTES* regulated on activation, normal T cell expressed and secreted, CCL5, *LTB4* leukotriene B4, *LTA4* leukotriene A4, *VCAM-1* vascular cell adhesion molecule 1 (CD106), *ICAM-1* intercellular adhesion molecule 1 (CD54), *HMGB-1* high-mobility group box 1 (belongs to danger-associated molecular patterns), *MCP-1* monocyte chemoattractant protein 1 (CCL2), *FoxP3* forkhead box P3, *CTL* cytotoxic T lymphocyte, *Th* T helper cell, *CTLA-4* cytotoxic T lymphocyte-associated protein 4 (CD152); CD39: nucleoside triphosphate diphosphohydrolase 1 (NTPD1); CD73: 5′-nucleotidase (5′-NT), *VEGF* vascular endothelial growth factor, *IDO* indoleamine-pyrrole 2,3-dioxygenase, *α-SMA* alpha smooth muscle actin, *CTGF* connective tissue growth factor (CCN2), *bFGF* basic fibroblast growth factor, *TCR* T cell receptor, *NFAT* nuclear factor of activated T cells, *NLRP3* Nod-like receptor family pyrin domain containing 3 gene, *ART2-P2X7 pathway* extracellular NAD^+^-induced ATP-independent p2X7R activation involving ADP-ribosyltransferase 2, *MMP-9* matrix metalloproteinase-9, *TIMP-1* tissue inhibitor of metalloproteinase 1, *LC-MS/MS* liquid chromatography and tandem mass spectrometry, *STAT-1* signal transducer and activator of transcription 1, *FPR* formyl-peptide receptor

### Purinergic regulation of the pro-inflammatory responses

**Fig. 3:**
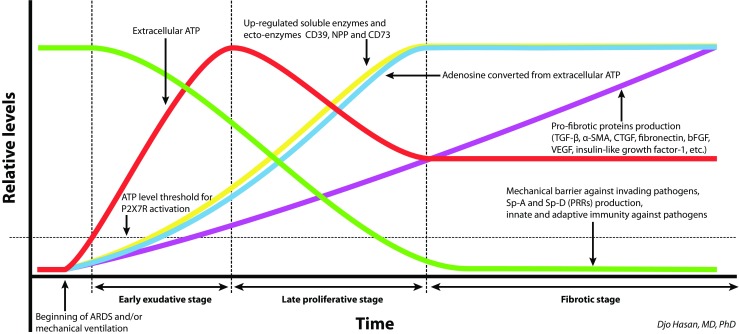
Putative model of local tissue purinergic signalling, pathogen barriers, adaptive immunity and pro-fibrotic proteins during ARDS and/or VILI [70]. In the very early phase of ARDS and VILI, infection [43, 52, 53] and vigorous mechanical deformation of the alveoli by mechanical ventilation [212, 213] lead to the substantial increase of extracellular ATP. The ATP levels exceed the threshold for the activation of P2X7R and induce pro-inflammatory immune response [70, 211]. This causes capillary congestion and capillary leakage causing interstitial and alveolar oedema. CD39 expression is upregulated in severe sepsis [214] and after several hours of mechanical ventilation [212]. Consequently, extracellular levels of ATP gradually decrease to a certain extent and extracellular adenosine increases. In general, adenosine has potent anti-inflammatory properties. This may lead to immune paralysis against secondary specific infections. Moreover, lung tissue damage due to DAD is accompanied by the disruption of the physical barrier as a component of the innate immunity for the defence against invading pathogens and by decreased Sp-A and Sp-D levels that function as soluble pattern recognition receptors (PRRs) of the innate immune system. This renders the host susceptible to invading pathogens [42]. TGF-β expression is increased by the activation of P2X7Rs [175, 176] and activation of the adenosine receptor AdoRA2B [132]. AdoRA2B activation also increases the expression of TGF-β and other fibrotic factors such as alpha smooth muscle actin (α-SMA), connective tissue growth factor (CTGF or CCN2), IL-6, fibronectin, VEGF, CD206, arginase-1, hyaluronan, basic fibroblast growth factor (bFGF), insulin-like factor-1, etc. (Table 3, rows 40, 44, 47 and 49) [128, 130, 132, 239]

Although many ATP receptors play a role in the activation of the pro-inflammatory immune response (Table 3), activated P2X7Rs are important initiators of the pro-inflammatory response by the innate immunity [43, 53, 210]. P2X7R is an ATP receptor and an intrinsic cation channel, but here, the P2X7R serves as an ATP receptor initiating intracellular transduction. Furthermore, the P2X7Rs have a low affinity for ATP molecules. They are activated only when the concentration of extracellular ATP is quite high reaching millimolar concentrations [70, 211], for example, during a severe inflammation leading to ARDS and/or vigorous mechanical deformation of the AT I cells (Fig. 3). In these conditions with high levels of extracellular ATP, the P2X7R does not appear to exhibit desensitization [64, 215]. Consequently, high levels of extracellular ATP molecules continuously act as DAMPs and activate the immune system [43, 53, 70]. In case of massive release, ATP may saturate the ATP hydrolysis enzymes CD39 and CD73 causing persistent high ATP levels despite the conversion to adenosine. The resulting pro-inflammatory immune response causes damage to the lung tissue and ends in DAD. Interstitial and alveolar oedema are the result of capillary leakage due to the pro-inflammatory response in the early exudative stage of DAD [34, 36].

Reportedly, another mechanism of the development of alveolar oedema in ARDS after influenza virus infection is proposed [216, 217]: influenza virus-induced disruption of the tight junctions between the alveolar epithelia cells and direct inhibition of amiloride-sensitive epithelial sodium channels (ENaCs) [217]. The devastation of the normal alveolar histology in DAD is such that the tight junctions between the epithelial cells are indeed disrupted. But massive ATP release by alveolar epithelial cells after H1N1 influenza A virus (IAV) [218, 219], respiratory syncytial virus (RSV) infection [220, 221] and parainfluenza infection virus infection [222] as detected by increased ATP in bronchoalveolar lavage fluid (BALF) have also been reported. Consequently, activation of P2X7Rs in epithelial cells and macrophages after a viral infection results in a pro-inflammatory response of the innate immune system [151] (Table 3, rows 63–66 and row 76) [162–164, 174] and lung tissue injury. Therefore, it is extremely difficult to assess whether the influenza-induced ENaC inhibition in the lung tissue plays a significant role in the development of alveolar oedema in ARDS with profound capillary leakage as part of the pro-inflammatory response and disrupted alveolar histology (DAD).

### Conversion of extracellular ATP to adenosine with anti-inflammatory properties.

After several hours of in vitro mechanical deformation of Calu-3 cells (a human airway epithelial cell line that shows cAMP-dependent Cl^−^ secretion [223]), the messenger RNA (mRNA) levels of CD39 and CD73 are upregulated [212]. Moreover, CD39 expression is upregulated in severe infection such as sepsis [214]. This is probably the consequence of increased TGF-β release due to the activation P2X7Rs by extracellular ATP (Table 3, row 77) [175, 176] and increased IFN-β [224–226] due to the activation of P2Y6Rs by extracellular ATP as presented in Table 3 row 92 [193]. Consequently, extracellular levels of ATP gradually decrease to a certain level, and extracellular adenosine increases. In general, adenosine has potent anti-inflammatory properties. Prolonged high levels of extracellular ATP lead to high levels of extracellular adenosine and to the secondary suppression of the innate and adaptive immune system (Fig. 3). The suppressive effects of extracellular adenosine on the adaptive immune system are presented in Table 3, rows 6–8 [81–84], 24–29 [106–112], 37 [125], 38 [126] and row 104 [206]. Note that adenosine promotes the differentiation of naïve T cells to CD4^+^FoxP3^+^Lag3^+^ Tregs [106], increases the numbers of Tregs [112], increases the expression of the co-inhibitory surface molecule CTLA-4 on Tregs [112] and upregulates the expression of CD39 and CD73 on Tregs [112]. Tregs are potent inhibitors of the activation and function of effector T cells and potent inhibitors of the maturation and function of DCs [227]. Moreover, Tregs reduce the survival of effector T cells [227]. This causes immune paralysis against secondary specific infections. Additionally, lung tissue damage due to DAD is accompanied by the disruption of the physical barrier, a component of the innate immunity for the defence against invading pathogens and by a decrease of certain soluble PRRs (SP-A and SP-D) production [41, 42].

### Intratracheal administered ATP or UTP molecules cause diffuse lung injury

Confirmation of the role of extracellular ATP in the pathogenesis of VILI is supplied by the following report by Matsuyama et al. [213]: Intratracheally ATP or UTP (100 and 200 mM) instilled mice showed a progressive increase in wet-to-dry (W/D) weight ratio of the lungs during the first 18 h, the extent of the increased W/D ratio was dose dependent. There was also a significant increase in the albumin permeability index. In comparison to the reference gene glyceraldehyde-3-phosphate dehydrogenase (GAPDH), ATP induced an increase in the gene expression (mRNA) of macrophage inflammatory protein-2 (MIP-2), TNF-α and IL-6 detected using real-time polymerase chain reaction (RT-PCR) within 60 min after the administration [213]. Note that the gene expression of IL-1β was not increased because ATP regulates the posttranscriptional activation of NLRP3 inflammasomes [152, 228, 229], and this cannot be detected with the applied RT-PCR technique. There was a considerable rise in the number of neutrophil and macrophage counts in the BALF. These effects appeared in intratracheally ATP instilled mice and in mice mechanically ventilated with a *V*_T_ of 40 ml/kg ideal body weight but not with a *V*_T_ of 8 ml/kg. The ATP levels in BALF specimens in the high *V*_T_ group were significantly increased when compared to the low *V*_T_ group. In addition, the increased W/D weight ratio, albumin permeability and cytokine production (with the exception of TNF-α) in the intratracheal ATP group were mitigated by the administration of pyridoxal-5′-phosphate-6-azophenyl-2′, 4′-disulfonic acid (PPADS), a semi-selective antagonist of the purinergic receptor P2X and P2Y purinergic receptors [213].

### Increased extracellular ATP levels to micromolar concentrations and conversion of very high levels of extracellular ATP (millimolar concentrations) to adenosine improve survival

Belete et al. applied five incremental steps of strain (0, 0.03, 0.06, 0.08, 0.10, 0.18, respectively) to experimental AT I cell monolayers [230]. Strain was defined as the proportion of radial length change of the monolayer during each stretch cycle. They found that the extent of the applied cell strain was proportional to the extracellular level of ATP and the extent of plasma membrane damage. Adding a small amount of ATP (10 μM) or ecto-enzyme inhibitor ARL 67156 (100 μM) decreased the proportion of the lethally wounded AT I cells. The addition of adenosine (5 μg/ml) did not provide increased cytoprotection. The addition of apyrase (20 U/ml) or apyrase combined with ADA (5 U/ml) to increase the extracellular adenosine levels at the expense of the extracellular ATP levels caused an increase in the proportion of the lethally wounded AT I cells ruling out a potential adenosynergic cytoprotection pathway. Apparently, elevated extracellular ATP levels to micromolar concentrations facilitated the plasma membrane wound healing by increasing the fusion of the calcium-sensitive lysosomes with the plasma membrane at the wound site. ATP induced this process in the AT I cells in an autocrine manner through the P2Y2Rs activating the IP_3_ and DAG/PKC pathways [230]. This process is similar to the paracrine ATP-induced fusion of the LBs with the cell membrane of the AT II cells [54, 55, 59].

Eckle et al. reported that the gene expression of the enzymes CD39 and CD73 that hydrolyse ATP to adenosine in the wild-type (WT) mice lungs was increased during mechanical ventilation with very high inspiratory pressure of 45 mbar (equivalent to 45.9 cm H_2_O) [212]. At this inspiratory pressure level, the lung volume reached TLC with a strain of 2.5 (strain was defined as the inspiratory volume divided by the functional residual capacity) [20, 231]. In CD39−/− and in CD73−/− knockout mice, diffuse lung injury was far more severe than in the WT mice [212]. Intraperitoneal administration of soluble CD39 in CD39−/− mice and soluble CD73 in CD73−/− mice attenuated the lung injury severity to the level of the WT mice [212]. In addition, intraperitoneal administration of soluble CD39 or CD73 in WT mice treated with the injurious mechanical ventilation increased the survival significantly, decreased the VILI score and increased the pulmonary adenosine levels [212]. As mentioned above, the very high strain levels cause a massive ATP release saturating the ATP hydrolysis enzymes CD39 and CD73 with persisting high ATP levels. The administered soluble CD39 and CD73 convert the excessive ATP molecules to adenosine causing the inflammatory ATP levels to drop and the anti-inflammatory adenosine levels to rise. In addition, adenosine attenuates the ventilator-induced capillary leakage through AdoRA2B receptor signalling (Table 3, row 43) [129].

As stated earlier, the ATP hydrolysing enzymes CD39 and CD73 are upregulated during mechanical ventilation. One of the factors that leads to the upregulation of CD73 is the increased expression of IFN-β [224–226] by the activation of P2Y6Rs [193]. Reportedly, intravenous administration of IFN-β-1a reduces mortality in patients with ARDS treated with assisted mechanical ventilation significantly [232].

### High levels of extracellular adenosine end in pulmonary fibrosis

On the other hand, when the extracellular ATP levels remain high due to continuous massive release of ATP, adenosine accumulates in the extracellular space due to the overload of the ADA enzyme and of the re-uptake process of adenosine through ENTs and CNTs (Fig. 2). Prolonged high levels of extracellular adenosine result in immunosuppression, the production of fibrotic proteins and finally end in fibrosis (Fig. 3). Reportedly, adenosine-dependent fibrosis occurs in mice with ADA deficiency. In these mice, ADA suppletion leads to the resolution of the fibrosis [233, 234]. On top of the increased TGF-β expression by the activation of P2X7Rs (Table 3, row 77) [175, 176], activation of AdoRA2B increases the expression of TGF-β, α-SMA, connective tissue growth factor (CTGF or CCN2), IL-6 and fibronectin in fibroblasts. These proteins are known to be pro-fibrotic (Table 3, row 47) (Fig. 3) [132]. One of the pro-fibrotic properties of TGF-β is the induction of epithelial-to-mesenchymal transition (EMT), a fundamental underlying pathogenic factor in lung fibrosis [235]. Presumably, treatment with IFN-β-1a, with soluble CD39 or with CD73, increases the adenosine levels. In turn, this probably leads to a significant decrease in extracellular ATP reducing the inflammation and the release of extracellular ATP by the lung tissue cells and the immune cells. For a proportion of patients [232] and experimental animals [212], this may well be enough to halt the progression of DAD.

## Summary and conclusion

**Fig. 4:**
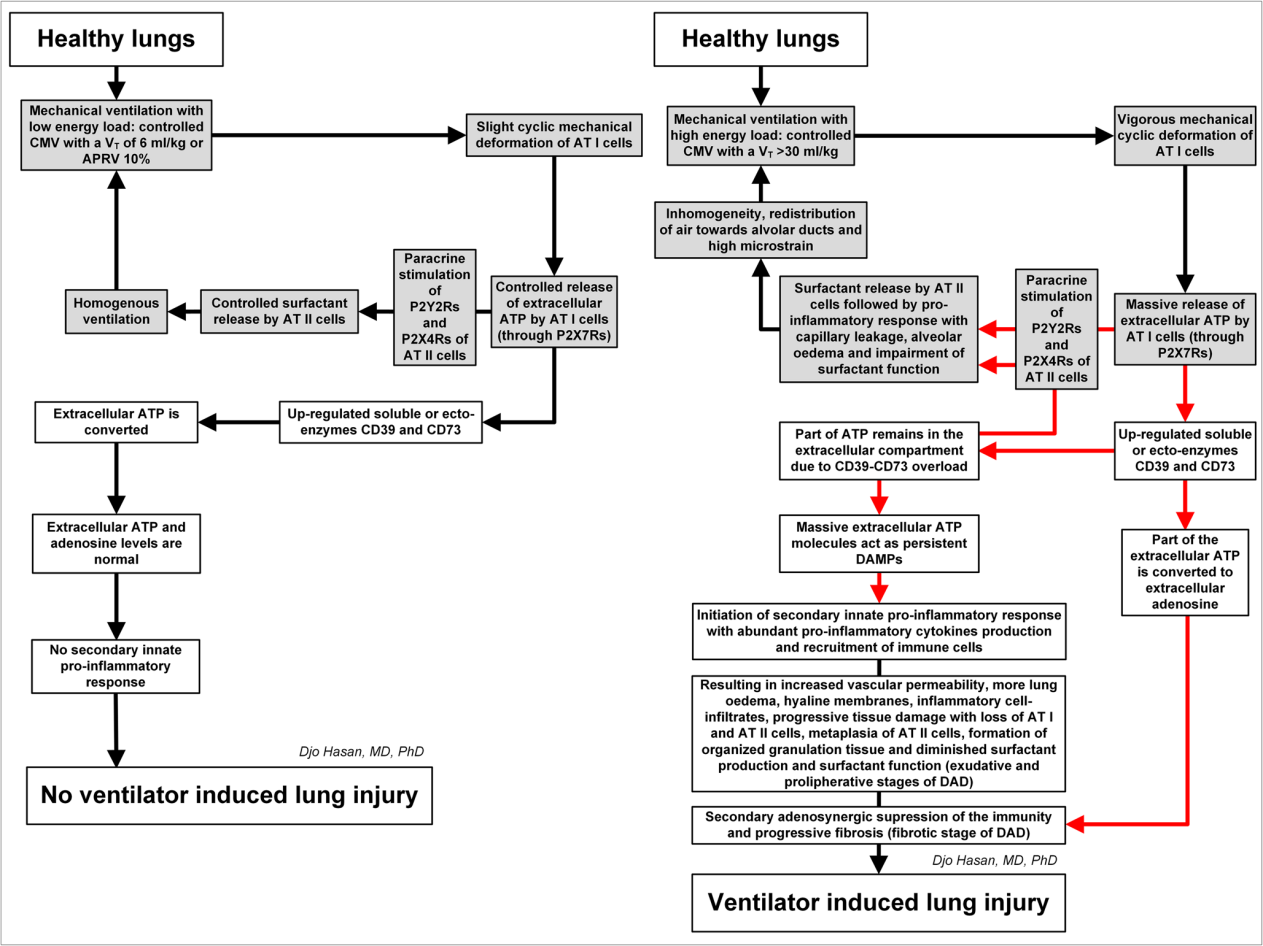
The summary of the physiological, pathophysiological and immunological consequences of controlled CMV with a *V*_T_ ≤ 6 ml/kg ideal body weight (*left*) and of controlled CMV with extremely high *V*_T_ (*right*) in healthy lungs (see text for explanation). The common cell signalling pathway for the release of surfactant by alveolar epithelial type 2 (AT II) cells and for the activation of the innate immunity (*red arrows*). Sequential processes related to mechanical ventilation (*grey coloured text boxes*). *CMV* continuous mandatory ventilation, *V*_T_ tidal volume, *APRV 10%* airway pressure release ventilation with the expiration termination set at 10% of the peak-expiratory flow rate (PEFR), *DAMPs* danger-associated molecular patterns, *DAD* diffuse alveolar damage

**Fig. 5:**
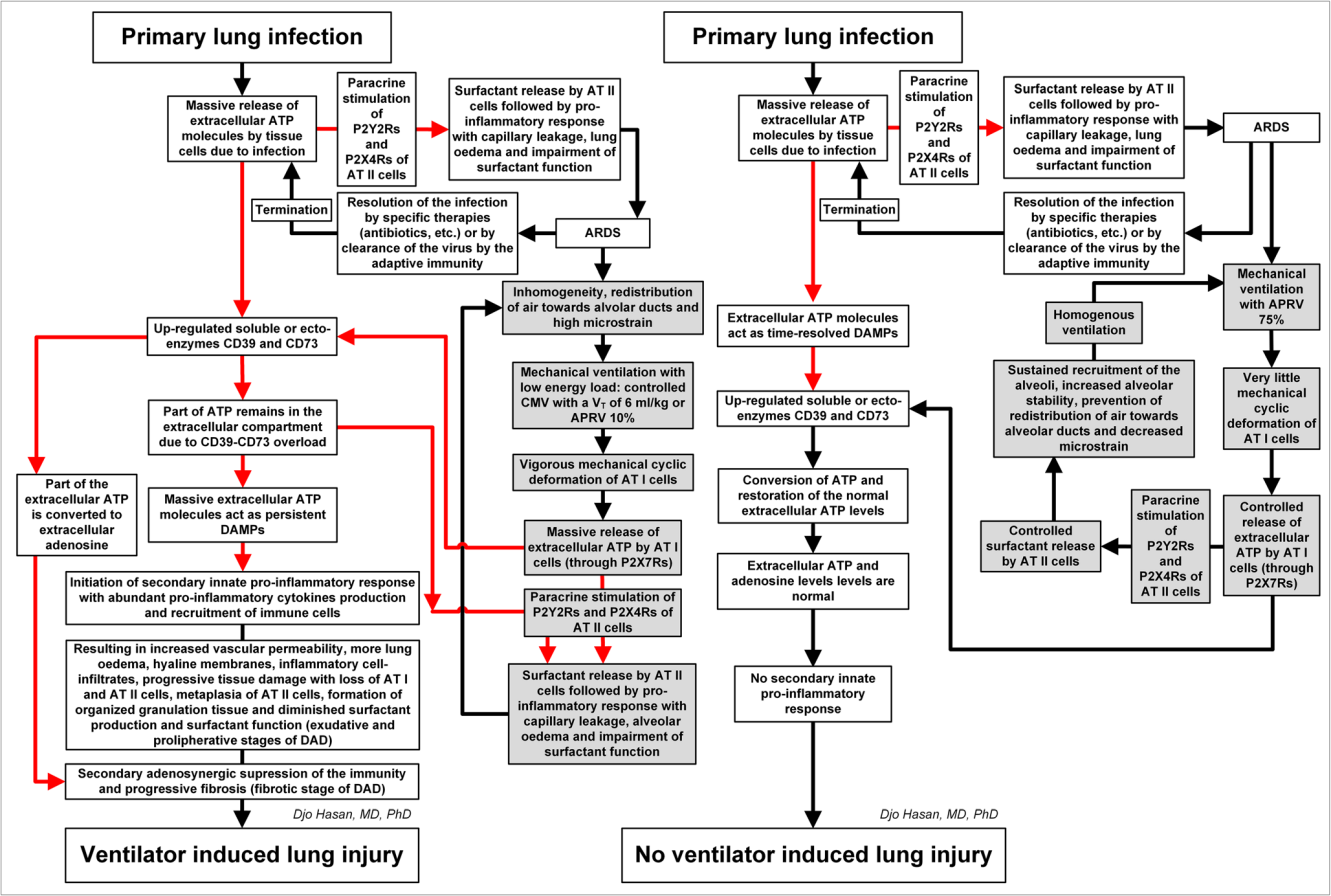
The summary of the physiological, pathophysiological and immunological consequences of controlled CMV with a *V*_T_ ≤ 6 ml/kg ideal body weight or APRV 10% (*left*) and of APRV 75% (*right*) in infected lungs (see text for explanation). The common cell signalling pathway for the release of surfactant by alveolar epithelial type 2 (AT II) cells and for the activation of the innate immunity (*red arrows*). Sequential processes related to mechanical ventilation (*grey coloured text boxes*). *CMV* continuous mandatory ventilation, *V*_T_ tidal volume, *APRV 75%* airway pressure release ventilation with the expiration termination set at 75% of the peak-expiratory flow rate (PEFR), *APRV 10%* APRV with the expiration termination set at 10% of the PEFR, *DAMPs* danger-associated molecular patterns, *DAD* diffuse alveolar damage

We summarise the physiological, pathophysiological and immunobiological consequences of controlled CMV, APRV 10% and APRV 75% in Figs. 4 and 5.

Diffuse lung tissue damage induced by systemic or local infection, trauma or haemorrhagic shock and mechanical ventilation all use a common cell signalling pathway to cause VILI. This pathway is as follows: AT I cells are the mechanosensors in the lung; the AT I cells secrete large quantities of ATP molecules after being infected or being vigorously stretched or compressed. This is followed by the paracrine stimulation of AT II cells by the extracellular ATP molecules leading to the unpacking of the LBs to the alveolar space. In infections, many infected immune cells and tissue cells (such as AT I and AT II cells) release ATP without requiring mechanical deformation of the cells.

Although increased extracellular ATP levels to micromolar concentrations may provide cytoprotection against stretched-induced plasma membrane damage by the facilitation of plasma membrane repair, very high levels (millimolar concentrations) of extracellular ATP activate the innate immune system by several other pathways: neutrophil recruitment and transmigration to the lung tissue, production of the pro-inflammatory cytokines IL-1β and IL-18, polarization of the macrophages towards M1 phenotype, etc. The resulting inflammation causes diffuse damage of the lung tissue and results in DAD. In addition, damage to the AT I and AT II cells (due to DAD) results in a decrease in surfactant production, extravasated serum proteins and the conversion of the active to the non-active surfactant subfraction ending in surfactant function impairment. The escalation of this process by ventilation (controlled CMV or APRV 10%) increases the extracellular ATP levels and surfactant impairment even further. Persistent release of extracellular ATP is followed by the upregulation of the ecto-enzymes (CD39 and CD73) decreasing extracellular ATP levels by conversion to adenosine. High levels of adenosine are associated with immune paralysis against invading pathogens and progressive lung fibrosis.

When the levels of the extracellular ATP are such that treatment with soluble CD39, CD73 or IFN-β is sufficient to reduce the pro-inflammatory response and restore the surfactant function, VILI development will be stopped and the lungs may recover. When the extent of lung damage is more severe and extensive with persistent high levels of extracellular ATP, conversion of extracellular ATP to adenosine does not lead to the recovery of the surfactant function but instead leads to very high levels of extracellular adenosine. In this case, VILI will progress to the next (fibrotic) stage (Fig. 3).

Mechanical ventilation by means of controlled CMV or APRV 10% with relatively long expiration time results in cyclic recruitment of the surfactant-deactivated alveoli causing a redistribution of the inspired air volume from the non-recruited alveoli to the alveolar ducts. In addition, cyclic recruitment leads to an increase in the volume difference between inspiration and expiration in the alveolar ducts and therefore increases the microstrain. Increased microstrain is associated with the increase of mechanical deformation of the alveoli causing an increase in extracellular ATP release. This ends in a vicious circle of mechanical tissue damage → massive ATP release → increased inflammation → surfactant function impairment → exacerbating mechanical tissue damage → massive ATP release → etc. (Figs. 4 (right panel) and 5 (left panel)).

The essence of the prevention of VILI is to escape this vicious circle by decreasing the extracellular levels of ATP in the lung tissue either by the administration of IFN-β [232], soluble CD39 and soluble CD73 [212] or by continuously recruiting the alveoli until all are recruited and to keep the recruited alveoli open [8]. This ventilator strategy prevents the redistribution of inspired air towards the alveolar ducts, stabilises the alveoli, minimises the microstrain and limits the release of extracellular ATP by the cells, especially by the AT I cells. Consequently, the innate immune response is reduced, and the AT I, AT II cells and surfactant function are restored. Properly set, APRV 75% appeared to do just that by opening the lung and keeping it open, in experimental settings [7 8 11] and in a clinical statistical analysis [9] (Fig. 5 (right panel)). The rationale behind the lung tissue protective effect of the APRV 75%-induced recruitment and stabilization in surfactant-impaired ARDS model in animals is through understanding dynamic alveolar mechanics. Alveoli are not elastic (i.e. change size in a one to one relationship with the applied stress, which would be the *V*_T_); rather, alveoli are a viscoelastic system. A viscoelastic system is represented best by the spring and dashpot [240]. In a viscoelastic system, there is a delay in the strain (i.e. change in alveolar size) following the applied stress, which in the case of the lung is the inspiratory pressure. At least two conditions have to be fulfilled to recruit the collapsed viscoelastic alveoli: (1) sufficiently high airway pressure of >30 cm H_2_O and (2) long enough duration of the pressure (varies between 2 s and several minutes for the individual alveolus due to the inhomogeneous nature of the collapsed alveoli) to allow the alveoli to be recruited [236, 237]. This is known as the creep phenomenon of the viscoelastic system [240]. Thus, by extending the time at inspiration (i.e. increasing the duration of the applied stress), we gradually ‘nudge’ alveoli open over time [236]. Fully recruiting the lung eliminates stress concentrators, a major VILI mechanism [238]. The lung is stabilised by a very short time at expiration. If the stress (i.e. inspiratory pressure) is released very quickly for a very short time, the viscoelastic alveoli will not have time to empty, maintaining a PEEP. Thus, the short expiratory duration stabilises the lung by two mechanisms: time and pressure. Using our understanding of dynamic alveolar mechanics and the fact that alveoli are viscoelastic, we use the MBP parameter of *duration* to open and stabilise the lung. Using a short expiratory duration stabilises alveoli and prevents the redistribution of the inspired air towards the alveolar ducts and prevents vigorous cyclic deformation of the alveoli [8], which, in turn, prevents massive extracellular ATP release, massive short-term surfactant release, followed by surfactant function impairment, interstitial and alveolar oedema and activation of the innate immunity leading to the development or aggravation of DAD. This presumably prevents the loss of AT I cells, prevents the hyperplasia of AT II cells and restores the surfactant function, and the affected lung regions start to regain their normal compliance (increase their alveolar time constant).

## Electronic supplementary material

ESM 1 (HTM 32.5 kb)

ESM 2 (HTM 32.5 kb)
